# Sediments as Potential Sources of Non‐Cyanobacterial Diazotrophs in Arctic Sea Ice and Seawater

**DOI:** 10.1111/1758-2229.70372

**Published:** 2026-06-04

**Authors:** Haitian Bo, Ziyuan Zhang, Amane Fujiwara, Yuri Fukai, Masato Ito, Satoshi Kimura, Marc Oggier, Hisatomo Waga, Laura Whitmore, Koji Hamasaki, Takuhei Shiozaki

**Affiliations:** ^1^ Atmosphere and Ocean Research Institute, the University of Tokyo Chiba Japan; ^2^ Research Institute for Global Change, Japan Agency for Marine‐Earth Science and Technology Yokosuka Japan; ^3^ Graduate School of Frontier Science, the University of Tokyo Chiba Japan; ^4^ International Arctic Research Center, University of Alaska Fairbanks Fairbanks Alaska USA; ^5^ International Polar and Earth Environmental Research Center National Institute of Polar Research Tokyo Japan

**Keywords:** Arctic Ocean, diazotrophs, sea ice, sediments, thermodesulfobacteriota

## Abstract

Although nitrogen fixation has recently been recognized in the Arctic Ocean, the biogeographical patterns of diazotrophs in this region remain poorly resolved, leaving a critical gap in understanding of environmental nitrogen inputs. To address these uncertainties, we examined diazotrophic and overall prokaryotic communities in first‐year land‐fast sea ice, seawater, and sediments of the Pacific Arctic to identify habitat‐specific patterns. Diazotrophic communities in sea ice and sediments exhibited stable structures across all samples, with both habitats consistently dominated by the anaerobic phylum Thermodesulfobacteriota (particularly Desulfuromonadia) and other sulfate‐reducing lineages. In contrast, seawater communities were spatially heterogeneous, with some samples showing elevated abundances of Thermodesulfobacteriota. Prokaryotic communities showed marked differences among habitats, with Thermodesulfobacteriota representing a major group in sediments. β‐diversity and amplicon sequence variant (ASV)‐sharing analyses revealed extensive overlap between sea ice and sediment diazotrophs. Given that sea ice is a transient habitat, these findings suggest that diazotrophs in sea ice originate from underlying sediments. Moreover, the Thermodesulfobacteriota ASVs detected in seawater were identical to those in sediments, indicating likely resuspension. Collectively, our results highlight sediments as key reservoirs of diazotrophs in the Pacific Arctic, particularly on the continental shelf.

## Graphical abstract

Diazotrophic communities across Arctic environments: Integrated analyses of diazotrophs in sea ice, seawater, and sediments show that Arctic continental shelf sediments function as a central reservoir, supplying diazotrophs to both sea ice and seawater through resuspension processes.

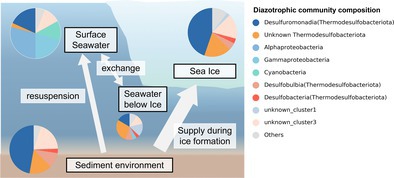

## 1 Introduction

Biological nitrogen fixation, the reduction of atmospheric N_2_ to ammonia by diazotrophs, is a primary source of bioavailable N in the ocean, sustaining new production (Zehr and Capone 2020). Marine nitrogen fixation has traditionally been attributed to photoautotrophic cyanobacteria, which were thought to be restricted to warm (> 20°C), oligotrophic, well‐lit tropical and subtropical waters of the global ocean (Sohm et al. 2011). However, recent evidence shows that many species of non‐cyanobacterial diazotrophs (NCDs) occur in diverse marine environments, including low‐latitude oceans (Farnelid et al. 2011; Delmont et al. 2022; Turk‐Kubo et al. 2023), polar oceans (Farnelid et al. 2011; Shiozaki et al. 2018, 2023; von Friesen, Laber, et al. 2025), deep‐sea waters (Hamersley et al. 2011; Gradoville et al. 2017), and sediments (Mehta and Barros 2006; Dong et al. 2022). NCDs are now recognized for their ability to adapt to various environments owing to diverse metabolic functions (Delmont et al. 2018, 2022; Shiozaki et al. 2023; Crétin et al. 2025; Deng et al. 2025).

In polar regions, research on nitrogen fixation has advanced rapidly, particularly in the Arctic Ocean. Since the first report of nitrogen fixation activity in the Arctic Ocean by Blais et al. (2012), studies have been conducted across the basin (von Friesen and Riemann 2020). In marginal regions of the Pacific Arctic, cyanobacterial diazotrophs, particularly the symbiotic lineage *Candidatus Atelocyanobacterium thalassa* (UCYN‐A), are frequently detected (Shiozaki et al. 2018, 2025; Harding et al. 2018; Cheung et al. 2022; von Friesen, Laber, et al. 2025), suggesting that UCYN‐A is transported to the Arctic from lower latitudes (Shiozaki et al. 2018, 2025). By contrast, NCDs are more commonly reported from the interior Arctic Ocean (Blais et al. 2012; Shiozaki et al. 2018; von Friesen, Laber, et al. 2025). Evidence of NCDs has been found not only in seawater but also in Arctic sediments (Jabir et al. 2021; von Friesen, Löscher, et al. 2025), as well as in sea ice and its brine channels (Díez et al. 2012; Fernández‐Méndez et al. 2016). Unlike cyanobacterial diazotrophs, the distributional characteristics of NCDs in the Arctic Ocean remain poorly understood. Their broader diversity and the scarcity and inconsistency of available data hinder a clear understanding of how NCDs are distributed across polar environments and how they integrate into the overall prokaryotic community.

Recent metagenomic analyses have revealed that some reconstructed diazotroph genomes from the Arctic Ocean are endemic (Shiozaki et al. 2023) and contain numerous glycosyltransferase genes. The presence of these genes suggests the ability to produce large amounts of extracellular polysaccharides, a feature typically observed among microbes with an attached lifestyle, such as those associated with sea ice and sediments (Ewert and Deming 2013; Casillo et al. 2018). Sea ice also entrains sediments as it forms (Darby et al. 2011; Eicken et al. 1997, 2000; Ito et al. 2019). We therefore hypothesize that diazotrophs in these two environments may be interconnected. Elucidating the potential connectivity of diazotrophs between sea ice and sediments is important for understanding ecological linkages among Arctic habitats and for placing diazotroph distribution in a broader regional context. To test this hypothesis, we collected sea ice, sediment, and seawater samples from the Pacific Arctic region to characterize the distribution of NCDs and evaluate their position within the prokaryotic community.

## 2 Experimental Procedures

### 2.1 Sea Ice and Seawater Sampling Around Pt. Barrow

**FIGURE 1:**
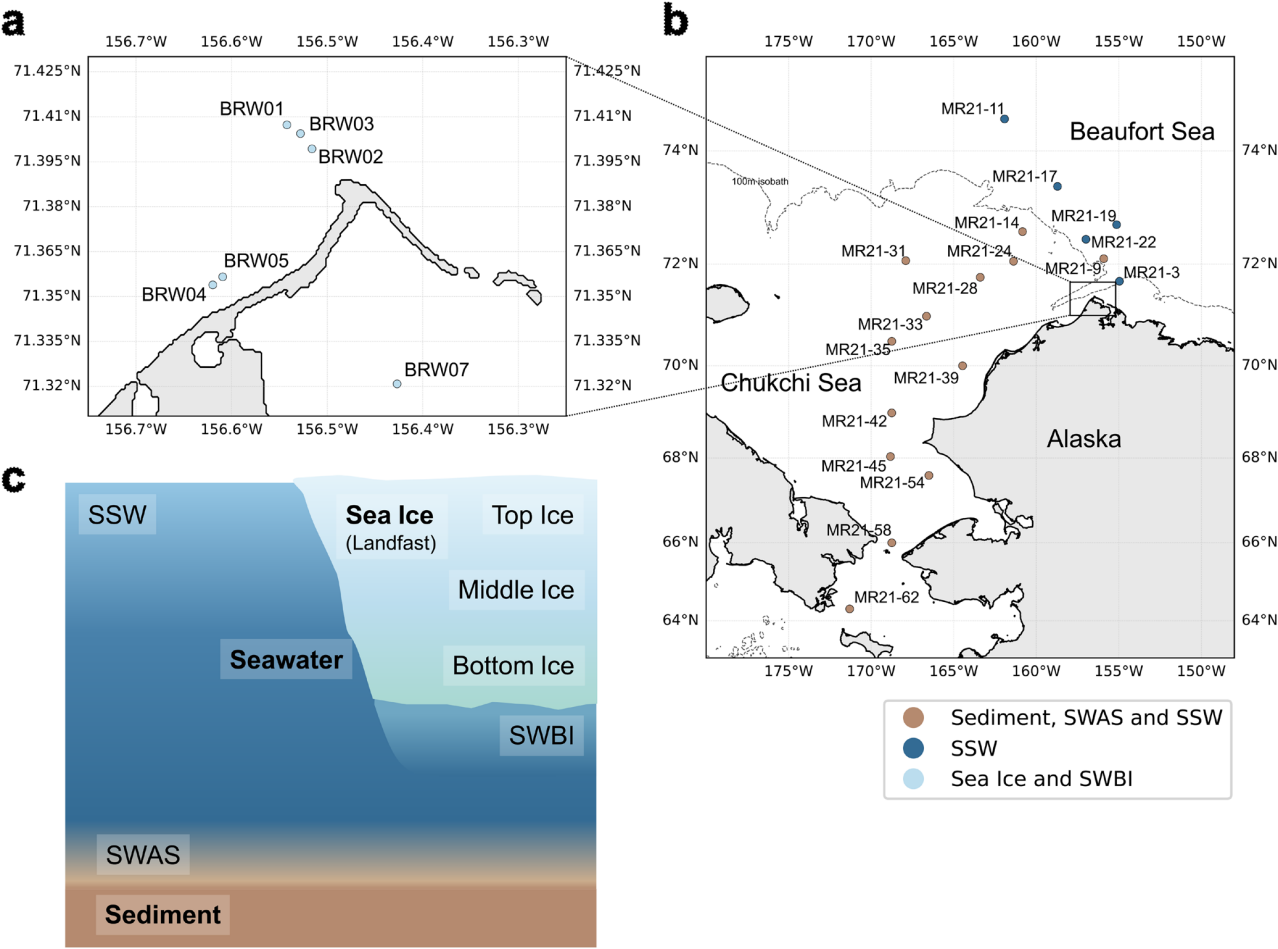
Sampling map and schematic overview of abbreviations. (a) Sampling sites for sea ice and under‐ice seawater (SWBI). The sea surface in this panel was fully covered by continuous landfast ice. (b) Sampling sites for sediment, seawater above sediment (SWAS), and surface seawater (SSW). The dashed line represents the 100 m isobath; areas below the line are shallower than 100 m, where most sediment samples were collected. (c) Conceptual diagram illustrating the sampling scheme and abbreviations. The schematic is not drawn to scale, and vertical distances between sampling layers may vary among sites. See Table S2 for exact depth. SSW: surface seawater; SWAS: seawater above sediment; SWBI: seawater below ice.

Sampling was performed from 12 to 15 May 2023 at six stations around Pt. Barrow, the northernmost point of Alaska, USA (Figure 1a). All of the sea ice in this region in May is considered first‐year ice (Petrich et al. 2012; Jones et al. 2016). Sea ice cores were collected from a continuous landfast first‐year sea ice cover using a Mark II ice coring system (Kovacs Enterprises Inc., Colorado Springs, CO, USA) with an internal diameter of 0.09 m and a length of 1.3–1.7 m. The landfast ice formed a stable, extensive ice sheet attached to the coastline, extending several kilometers offshore, rather than isolated drifting ice floes.

Each core was collected from a different location on the ice sheet. At each sampling site, a single core was extracted vertically from the ice surface downward. To obtain the full core length (1.3–1.7 m), coring was conducted in two steps: an initial core (~1 m) was first retrieved, followed by a second coring after attaching an extension rod to recover the remaining lower section. The two sections were combined to reconstruct the full core length. After extraction, the cores were sectioned on‐site, using an ice saw, into three layers: top (uppermost 10 cm from the surface), middle (10 cm centered at half of the total core length), and bottom (lowermost 10 cm from seawater). Each section was placed in an individual plastic bag and sealed for preservation. Upon return to the onshore laboratory of Utqiaġvik (formerly known as Barrow), the outer surface of the cores was carefully removed with a clean stainless‐steel knife to minimize potential contamination from surface‐adhering materials and then immediately melted in a water bath.

Seawater samples were also collected from beneath the ice through the coring hole (hereafter, SWBI; Figure 1c) using a water pump and stored in polycarbonate bottles for subsequent processing. After removal of the ice core, seawater partially filled the hole; to ensure sampling of under‐ice seawater, the intake tubing was inserted to the ice–seawater interface depth before pumping. Temperature and salinity of seawater were measured on site using a RINKO Profiler (JFE Advantech Co. Ltd., Hyogo, Japan).

Seawater and melted sea ice samples for nutrient analysis were collected in duplicate in 10 mL acrylic tubes and frozen at −20°C until further analysis. Samples for DNA analysis were filtered using cellulose acetate filters with a 0.22 μm pore size (Merck Millipore, Burlington, MA, USA) and stored frozen at −20°C until further analysis.

### 2.2 Sediment and Seawater Sampling in the Chukchi Sea

Sampling was performed aboard the R/V *Mirai* (MR21‐05C cruise) in the western Arctic Ocean from 15 September to 2 October 2021. The sampling stations broadly covered the Chukchi shelf region (Figure 1b). Sediment was sampled using a G.S. type corer (Asyura, RIGO Co., Tokyo, Japan), equipped with a polycarbonate core tube (ϕ 8.2 cm × 60 cm). The overlying seawater was carefully siphoned off, and a portion was collected for DNA analysis (hereafter, SWAS; Figure 1c). Surface sediments for DNA analysis were scraped with a sterilized spoon, placed into 25 mL tubes, and stored in a freezer at −20°C.

Seawater samples were collected at all stations from the surface using a bucket and from near the bottom using Niskin‐X bottles. Temperature, salinity, and dissolved oxygen profiles were continuously measured using the SBE911 plus CTD system (Sea‐Bird Electronics, Bellevue, WA, USA). Seawater samples for nutrient analysis were collected in duplicate in 10 mL acrylic tubes and analyzed immediately on board. Those for DNA analysis were collected from the surface (hereafter, SSW; Figure 1c) into 2.3 L polycarbonate bottles and from just above the sediments into 3 L polycarbonate bottles, then filtered using Sterivex‐GP pressure filter units with a 0.22 μm pore size (Millipore). The filters were stored frozen at −20°C until further analysis.

### 2.3 Nutrients

For sea ice and SWBI samples collected around Pt. Barrow, nitrate, nitrite, ammonium, and phosphate concentrations were measured in the laboratory using an AACSIII autoanalyser (Bran+Luebbe, Norderstedt, Germany). Detection limits (0.4, 0.02, 0.04, and 0.04 μM, respectively) were defined as twice the standard deviation of replicate blank measurements. For seawater samples collected from the Chukchi Sea, these were determined on board using the QuAAtro 2‐HR system (SEAL Analytical, Southampton, UK), with detection limits of 0.03, 0.005, 0.04, and 0.01 μM, respectively.

### 2.4 Molecular Analyses and Sequencing

Environmental DNA was extracted from sea ice and SWBI samples collected around Pt. Barrow, as well as from sediment and seawater samples collected from the Chukchi Sea. Filter samples containing sediment particles and bulk sediment samples were processed using the PowerSoil Pro Kit (QIAGEN, Hilden, Germany), while the remaining filter samples were processed with the DNeasy Plant Mini Kit (QIAGEN), following the manufacturer's instructions. DNA concentrations were quantified using a Quantus Fluorometer (Promega, Madison, WI, USA).

To assess the characteristics of both the diazotroph and overall prokaryotic communities, amplicon sequencing analysis targeting the *nifH* and 16S rRNA sequences was performed. For *nifH*, fragments were amplified using a nested polymerase chain reaction (PCR) approach (Zehr and Turner 2001). First‐round PCR was performed with the primers nifH3 and nifH4 (Zehr and Turner 2001). In the second round, nifH1 and nifH2 primers (Zehr and Turner 2001) with adapter sequences for Illumina sequencing were used. For 16S rRNA, primers 515F and 806R, with adapter sequences for Illumina sequencing, were used to amplify the V4 region (Caporaso et al. 2011). PCR conditions are provided in Table S1. All PCR reactions were performed using KAPA HiFi HotStart Ready Mix (KAPA Biosystems, Boston, MA, USA). PCR products were purified using the AMPure XP Purification Kit (Beckman Coulter, Brea, CA, USA).

Then, index PCR was performed using the KAPA HiFi HotStart Ready Mix to append the 7‐base barcode using KAPA HiFi HotStart Ready Mix (KAPA Biosystems). Purified products were quantified, and all samples from the same sampling period were pooled after concentration adjustment. Libraries were separately sequenced on an Illumina MiSeq platform (Promega) using the MiSeq v3 kit (600 cycles; Illumina) with the Phix control v3 (Illumina) for 300 bp paired‐end reads.

### 2.5 Sequence Data Processing and Analyses

After obtaining the sequence data, adapter sequences were removed using cutadapt (Martin 2011). Then, the data were processed with QIIME2 version 2022.8 (Bolyen et al. 2019). Following quality control, primer sequences and low‐quality fragments were removed. DADA2 version 1.22.0 (Callahan et al. 2016) was used to filter low quality sequences, denoising reads, generating amplicon sequence variants (ASVs), and identifying representative sequences (trimming parameters: 230 bp for forward and reverse reads of 16S rRNA sequences; 190 bp for forward and reverse reads of *nifH* sequences).

For 16S rRNA, 8476 ASVs were obtained, with read counts per sample ranging from 16,225 to 154,185. The Silva reference database, release 138, was used to assign taxonomy to both the eukaryotic and prokaryotic data sets (Quast et al. 2012). After removing sequences classified as chloroplast and mitochondria, read counts were normalized to the smallest value without discarding any samples. Rarefaction curves indicated that all samples reached asymptotes. Relative abundances were calculated and visualized at multiple taxonomic levels (phylum, class, order, family, genus).

For *nifH*, 5758 ASVs were obtained, with read counts per sample ranging from 4253 to 172,338. Rarefaction curves indicated that all samples reached asymptotes, and read counts were normalized to the smallest value without discarding any samples. The data were translated into amino acid sequences, and non‐*nifH* and frameshifted sequences were excluded. Then, the sequences were assigned to clusters based on the *nifH* database (v1.1.0; Moynihan 2020). *nifH* amino acid ASVs were assigned to the canonical *nifH* clusters (Zehr et al. 2003) using a BLASTp search against a curated reference database consisting of 879 publicly available full‐length *nifH* sequences (https://www.zehr.pmc.ucsc.edu/Genome879).

Statistical analyses were conducted in R (v. 4.3.2) and Python (v. 3.9.13). β‐diversity was analysed using log_10_(*x* + 1)‐transformed ASV abundance tables with Bray‐Curtis dissimilarities and non‐metric multidimensional scaling (NMDS; Kruskal 1978). Group differences in community structure were tested using permutational multivariate analysis of variance (PERMANOVA) (999 permutations), implemented with the metaMDS and adonis functions of the vegan package (v. 2.6–4; Oksanen et al. 2022). UpSet analysis was performed after filtering out ASVs with fewer than five reads in a sample. Community overlap analysis was quantified using the Jaccard index based on presence‐absence data for abundant ASVs only, defined as those present in at least two samples from one environment and represented by at least 100 reads in the dataset.

## 3 Results

### 3.1 Environmental Settings

Across sea ice stations, salinity ranged from 3.1 to 8.0 within the ice, with values of 4.3–6.8 in the top layer, 3.1–5.2 in the middle layer, and 5.3–8.0 in the bottom layer. In contrast, SWBI salinity remained stable, ranging only from 32.8 to 33.2 (Table S1). The temperature of the sea ice ranged from −6.19°C to −3.40°C, while the temperature of SWBI was from −1.95°C to −1.92°C.

Within the ice matrix, top and middle ice contained comparable concentrations of nitrate and ammonium (top ice: NO_3_^−^ 0.40–1.63 μM, NH_4_^+^ 0.52–1.68 μM; middle ice: NO_3_^−^ 0.47–1.66 μM, NH_4_^+^ 0.39–1.19 μM), with low phosphate (PO_4_^3−^ 0.076–0.247 μM), whereas the concentrations were elevated in the bottom ice (NO_3_^−^ 2.37–13.76 μM; NH_4_^+^ 0.64–4.45 μM; PO_4_^3−^ 0.45–4.63 μM). The SWBI was nitrate‐dominated (NO_3_^−^ 5.18–12.04 μM; NO_2_^−^ 0.043–0.099 μM; NH_4_^+^ 1.84–2.57 μM; PO_4_^3−^ 0.99–1.69 μM). Inorganic N/P ratios in both sea ice and SWBI (2.3–15.3, ~6.7) were generally below the Redfield ratio (16) (Figure S1).

The salinity values of sea ice observed in our study (3.1–8.0) fall within the range of past observations (3.2–13.3; Deming et al. 2020). In addition, nutrient concentrations were higher in bottom ice than in overlying layers, in line with previous findings (Deming et al. 2020). These results indicate that the sea ice sampled in our study reflects typical conditions for this region.

During the cruise period in the Chukchi Sea, salinity in SSW ranged from 25.89 to 32.10, while near‐bottom values were 31.32–32.95. Temperatures ranged from −1.17°C to 5.13°C in SSW and from −1.59°C to 4.95°C in near‐bottom waters. The SSW was generally N‐poor (NO_3_^−^ frequently not detected to 0.45 μM; NH_4_^+^ 0.04–0.69 μM), except at a few stations where concentrations were elevated (NO_3_^−^ 1.62–7.76 μM; NH_4_^+^ 0.68–2.09 μM). In contrast, near‐bottom waters were consistently nutrient‐replete (NO_3_^−^ 1.42–19.94 μM; NH_4_^+^ 0.07–9.41 μM; PO_4_^3−^ 0.94–2.71 μM). Inorganic N/P ratios in all seawater samples were below the Redfield ratio (0.04–13.34; average 4.29).

### 3.2 Prokaryotic Communities

**FIGURE 2:**
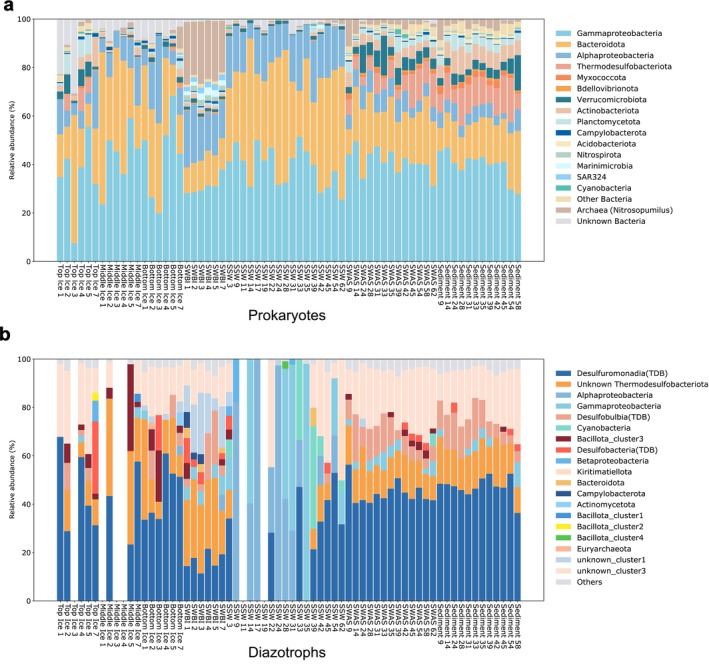
Relative abundance of diazotrophic communities (a) and prokaryotic communities (b). Members are grouped at the phylum level, except for Proteobacteria (class level) in both communities and Thermodesulfobacteriota (TDB; class level) in diazotrophic communities. Blank bars in (b) indicate samples in which no nifH gene amplification was detected.

The composition of prokaryotic communities in sea ice varied according to the ice layer (Figure 2a). Although the three layers shared similar community compositions dominated by Gammaproteobacteria (35.2%, 43.4%, and 43.5% on average for the top, middle, and bottom layers, respectively), Bacteroidota (22.0%, 36.2%, and 27.5%) and Alphaproteobacteria (10.2%, 10.7%, and 13.9%) also constituted major fractions of the communities. In addition, compared with the middle and bottom layers, the top ice was characterized by relatively higher proportions of Thermodesulfobacteriota (3.8%, 0.2%, and 0.6%), Verrucomicrobiota (4.4%, 0.4%, and 1.6%), and Planctomycetota (4.1%, 0.4%, and 0.9%). A clear shift in community composition was observed from bottom ice to SWBI. SWBI was characterized by a markedly higher archaeal fraction (24.0%), largely composed of the genus Nitrosopumilus. Moreover, Nitrospirota, Marinimicrobia, and SAR324 were present in all SWBI samples (2.2%, 2.4%, and 2.2%, respectively).

Prokaryotic communities in SSW exhibited no marked variation among stations, mainly composed of Gammaproteobacteria, Bacteroidota, and Alphaproteobacteria, with a very low archaeal fraction (0.4%) and lacked detectable Thermodesulfobacteriota.

Compared to SSW, SWAS communities had higher proportions of Thermodesulfobacteriota, Verrucomicrobiota, Actinobacteria, Planctomycetota, and Archaea. No marked differences were observed between the communities in SWAS and those in the sediment.

**FIGURE 3:**
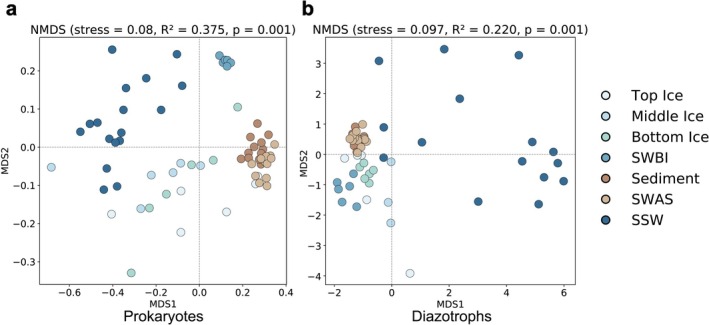
nMDS ordination plots for samples with (a) prokaryotic and (b) diazotrophic communities.

NMDS (Figure 3a, PERMANOVA *R* ^2^ = 0.418, *p* = 0.001) divided the prokaryotic communities into four groups: sea ice, SWBI, SSW, and sediment environment. The SWBI communities formed a distinct cluster, separate from both SSW and sea ice. Sea ice samples clustered between sediment and SSW, with some top ice samples forming a close cluster with sediment samples.

**FIGURE 4:**
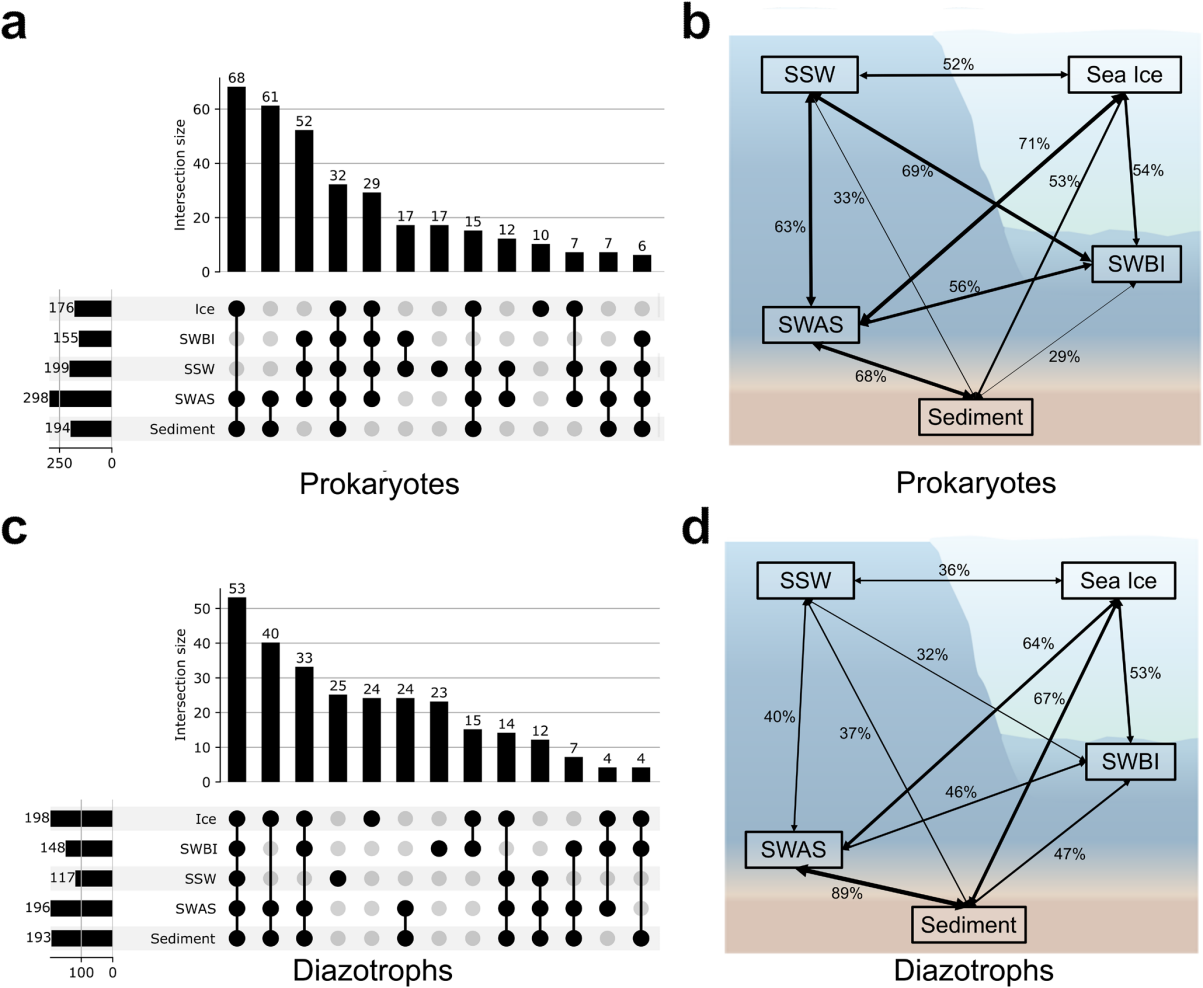
UpSet plot (a, c) and community overlap diagrams (b, d) for 16S rRNA ASVs (a, b) and *nifH* ASVs (c, d). In the UpSet plot, vertical bars (top) indicate the number of ASVs in each intersection, while the connected dots below show the contributing sample type. Line thickness in the overlap diagrams reflects the percentage of shared ASVs among communities.

We further examined the occurrence of the 300 most abundant 16S ASVs to compare the prokaryotic community across habitats at higher resolution (Figure 4a). An UpSet plot revealed shared ASVs among different environments: among the top 300 ASVs, 68 were shared between sea ice and sediment environments, 61 were shared between sea ice and SSW, and 52 were shared by all three water types. These patterns suggest that the overall prokaryotic community was broadly mixed among habitats.

Community overlap analysis (Figure 4b) indicated that, in addition to the high similarity between sediment and SWAS prokaryotic communities (68%), microbial communities among different water masses also exhibited relatively high similarity (SWBI–SSW, 69%; SSW–SWAS, 63%). In addition, sea ice communities showed substantial similarity with both SSW and SWBI (52% and 54%, respectively), as well as with sediment communities (56%), consistent with the extensive overlap revealed by the UpSet analysis.

Together, these results indicate extensive overlap of prokaryotic communities across habitats. The observed similarity was not confined to any single habitat pair, but involved sea ice, multiple water masses, and sediment, suggesting broad exchange and mixing of the overall prokaryotic microbiome within this coastal Arctic system.

### 3.3 Diazotrophic Communities

The *nifH* gene was also successfully recovered from all environments studied, including seawater, sea ice, and sediments, but not from all individual samples; several samples showed no detectable *nifH* amplification (Figure 2b).

Diazotroph communities in sea ice exhibited a consistent composition at the phylum/class level based on the *nifH* profiles across ice layers: Cluster 3 was dominant (~55%/59%/54% for top/middle/bottom), followed by Cluster 1 (~45%/41%/46%); Proteobacteria prevailed overall, with Thermodesulfobacteriota sublineages making up the major fraction (64.8%–74.0%). Within Thermodesulfobacteriota, Desulfuromonadia formed the core community across layers (Cluster 1, ~45.4%/41.4%/44.8% for top/middle/bottom), accompanied by variable proportions of unassigned Thermodesulfobacteriota (Cluster 3, ~8.9%/32.4%/18.0%). Desulfobulbia (Cluster 3) were secondary but were elevated in individual samples at station BRW02 and BRW05. Cyanobacteria were consistently rare in *nifH* annotations (≤ 0.14%).

In SWBI, Thermodesulfobacteriota remained predominant, although the relative proportions of their sublineages differed from those in sea ice. Cluster 3 accounted for an even higher proportion (~61%). Unassigned Thermodesulfobacteriota were more abundant (Cluster 3, ~28.5%), followed by Desulfuromonadia (Cluster 1, ~16.5%) and Desulfobulbia (Cluster 3, ~8.9%). Unclassified assignments were also substantial (top/middle/bottom ice/SWBI = 26.6%/8.8%/19.1%/27.8%).

The diazotroph community structure in SSW was markedly different from that in sea ice, sediments, and associated seawater, and was dominated by Cluster 1 *nifH* (~80%). In most samples, the major components consisted of Alphaproteobacteria, Gammaproteobacteria, and Cyanobacteria.

The diazotroph community structure in SWAS and sediments showed no obvious differences: Cluster 1 and 3 contributed nearly equally (~50%/47% and 50%/53% in sediment/SWAS, respectively); Thermodesulfobacteriota dominated both habitats. Within Thermodesulfobacteriota, Desulfuromonadia consistently accounted for the largest proportion (Cluster 1, SWAS: ~44.6%; sediments: ~47.2%), followed by unassigned Thermodesulfobacteriota (Cluster 3, ~13.5%–15.2%) and Desulfobulbia (Cluster 3, ~9.4%–20%). A substantial fraction of the community remained unclassified (SWAS/sediments = 23.1%/19.9%).

Beta‐diversity analysis (Figure 3b) revealed clear clustering of sea ice and sediment samples, distinct from the more heterogeneous seawater communities. PERMANOVA confirmed significant differences among habitats (*R* ^2^ = 0.22, *p* = 0.001), with sea ice and sediments exhibiting greater similarity to each other than to seawater.

An UpSet plot of the 300 most abundant *nifH* ASVs (Figure 4c) showed clearer distribution patterns compared to 16S rRNA ASVs. Of the top 300, 53 ASVs were shared across all environment types, while 73 were shared specifically between sediments and sea ice, along with numerous ASVs detected in only a single environment type.

Community overlap analysis (Figure 4c) further highlighted contrasting similarity patterns for *nifH* ASVs compared to 16S rRNA ASVs. Sea ice and sediment were highly similar (67%), substantially more so than sea ice and SSW (36%) and the different seawater environments (SWBI–SSW, 32%; SSW–SWAS, 40%). These patterns were consistent with the results of the UpSet analysis.

Together, these results indicate that, unlike the broadly mixed pattern observed for the overall prokaryotic community, diazotroph community showed a more structured distribution across habitats, with particularly strong connectivity between sea ice and sediment.

## 4 Discussion

### 4.1 Prokaryotic Communities in Sea Ice, Surface Seawater, and Sediment

Knowledge of prokaryotic communities within Arctic sea ice remains limited (Bowman et al. 2012; Bowman 2015; Torstensson et al. 2021; Yergeau et al. 2017; Hatam et al. 2016). Bowman et al. (2012) and Torstensson et al. (2021) reported that multiyear ice communities in the central Arctic Ocean are dominated by Gammaproteobacteria, together with Bacteriodota (formerly classified as Flavobacteria), Cyanobacteria, and Alphaproteobacteria. In the Canadian Arctic Archipelago, Gammaproteobacteria and Bacteroidota are the dominant taxa, while Alphaproteobacteria and Deltaproteobacteria, Planctomycetes, and Firmicutes occur in lower but notable proportions (Yergeau et al. 2017).

The first‐year ice community observed in this study was broadly similar to those reported previously. We observed vertical variation in community composition within sea ice, which may reflect the process of sea‐ice growth. Because sea ice develops downward after its initial formation (Tucker III et al. 1999), microorganisms can be incorporated from the surrounding environment at different stages of ice growth, potentially leading to distinct communities among layers. We further found that the SWBI community displayed several distinctive characteristics. Most notably, the relative abundance of nitrification‐related microorganisms, including *Nitrosopumilus* and Nitrospirota, was elevated. Of these, *Nitrosopumilus* is known to be sensitive to light inhibition of nitrification (Merbt et al. 2012). Because SWBI is expected to experience lower light availability than the SSW in the free‐ice region, this difference may partly explain the higher relative abundance of *Nitrosopumilus* in SWBI.

By contrast, numerous studies have examined prokaryotic communities in the Arctic Ocean, particularly in seawater, with many focusing on the Chukchi Sea (Comeau et al. 2011; Han et al. 2015; Kirchman et al. 2010). These studies have consistently reported that surface seawater prokaryotic communities in this region are dominated by Bacteroidota, Gammaproteobacteria, and Alphaproteobacteria. The composition of the SSW community in this study was generally consistent with these previous reports. In recent years, benthic microbial communities in the Chukchi Sea have also received increasing attention. Surveys by Dong et al. (2015, 2017), Sun et al. (2022), Walker et al. (2023), and Xie et al. (2024) reported broadly consistent community structures dominated by Proteobacteria (mainly Gammaproteobacteria and Alphaproteobacteria), together with Bacteroidota, Verrucomicrobiota, Actinobacteriota, and Planctomycetota, while Thermodesulfobacteriota typically accounted for 10%–20% of total sequences. In the present study, community compositions of sediment environments were also generally consistent with those reported previously, as we likewise detected a relatively stable Thermodesulfobacteriota assemblage. These results indicate that the SSW and sediment communities largely followed the previously recognized community patterns of the Chukchi Sea and also provided an important background reference for the differences observed in sea ice and seawater below ice.

### 4.2 Diazotrophic Communities in Sea Ice, Surface Seawater, and Sediment

In this study, DNA samples were collected using a 0.22 μm filter. However, recent studies have demonstrated the occurrence of diazotrophs smaller than 0.2 μm in the Arctic Ocean (Pierella Karlusich et al. 2021; Shiozaki et al. 2023). Accordingly, it should be noted that such diazotrophs may have been overlooked in the present study.

Previous work on Arctic sea ice diazotrophs has been extremely limited, and because those studies relied on sequencing based on clone libraries, they provided only a partial view of community structure (Díez et al. 2012; Fernández‐Méndez et al. 2016). Díez et al. (2012) reported abundant cyanobacterial *nifH* sequences from sea ice, but their primer set differed from ours and from other *nifH* studies in the Arctic Ocean, making direct comparisons difficult. Fernández‐Méndez et al. (2016) found that Gammaproteobacteria and Alpha/Betaproteobacteria were abundant in upper ice of the central Arctic Ocean, whereas Thermodesulfobacteriota (formerly classified as Deltaproteobacteria) and other cluster III lineages were more common in bottom ice. Our findings partially overlap with these observations. We identified Desulfuromonadia (cluster I Thermodesulfobacteriota), cluster III Thermodesulfobacteriota, and unassigned bacterial lineages across different sea ice layers.

The diazotrophic community structure in surface seawater from our study region has been described in detail by Shiozaki et al. (2018, 2025). They demonstrated that community composition varied considerably across sampling sites, and in some cases was dominated by a single species. Our results are in line with this pattern. Diazotrophic communities in sediments have also been investigated recently, in Svalbard fjords and in bathypelagic regions of the central Arctic Ocean (Jabir et al. 2021; von Friesen, Laber, et al. 2025). Jabir et al. (2021) reported that Deltaproteobacteria dominated *nifH* amplicons from Svalbard fjord sediments, with Desulfuromonadaceae, Desulfovibrionaceae, and Desulfobacteraceae as major contributors. Likewise, von Friesen, Laber, et al. (2025) highlighted the dominance of Desulfuromonadia in bathypelagic sediments. Despite differences in setting, with our samples from the Chukchi Sea shelf and theirs from fjords and deep‐sea sites, all studies converge on the prominence of sulfate‐reducing diazotrophs in Arctic sediments.

### 4.3 Potential Source of Diazotrophs in Sea Ice and Seawater

In this study, we characterized diazotrophic communities in both sea ice and sediments of the Chukchi Sea. In line with previous work, our results indicate that diazotrophs are broadly distributed across Arctic habitats, including seawater, sea ice, and sediments. Although near‐bottom waters contained relatively high concentrations of inorganic N nutrients, all seawater and sea ice environments showed N/P ratios below the Redfield ratio, indicating relative N limitation. Also, previous research has reported low sediment N/P ratios of 1.4–3.4 in the same region (Zhang et al. 2010). These relatively N‐deficient conditions might create ecological niches favorable for diazotrophs. The diazotrophic community structure in sea ice and sediments was remarkably similar: NCDs predominated in both environments, with Thermodesulfobacteriota being the dominant group.

Thermodesulfobacteriota are generally recognized as anaerobic bacteria. Marine sediments readily develop anaerobic conditions, thereby supporting substantial populations of anaerobic microorganisms (Jørgensen et al. 2019, 2022). Our observations are consistent with this expectation. Anaerobic microenvironments also occur within sea ice, where brine channels become oxygen‐depleted due to intense aerobic respiration combined with restricted gas exchange (Rysgaard and Glud 2004; Rysgaard et al. 2008). Consequently, both sediments and sea ice provide anaerobic niches that likely facilitate the persistence of Thermodesulfobacteriota in these two habitats.

The origin of diazotrophs in sea ice remains an important question. Because sea ice is a temporary habitat, its microbial inhabitants must be supplied from surrounding environments (Lund‐Hansen et al. 2024). Our results show that the diazotrophic community structure in sea ice closely resembled that in sediments, implying that sediments may be a major source of diazotrophs entrained into forming ice. Sea ice naturally incorporates sediments during its growth. In coastal regions, growing ice can contact the seafloor and shorelines, scraping and entraining fine sediment particles into brine channels and micropores within the ice matrix (Darby et al. 2011; Eicken et al. 1997, 2000; Ito et al. 2019), thereby incorporating sediment‐associated microbial communities. It should be noted that the sea ice and sediment samples in this study were collected in different years (2023 and 2021, respectively). However, previous studies have shown that microbial communities in Arctic surface sediments can remain relatively stable over time, including across seasonal transitions (Miksch et al. 2021, 2024). This suggests that surface sediments may act as a relatively stable microbial reservoir, in contrast to seawater communities that can vary more strongly in space and time. Seawater could also contribute to community structure in sea ice; however, diazotrophic communities in SSW varied substantially among stations. Previous studies have likewise demonstrated pronounced spatial and temporal heterogeneity in SSW diazotroph communities (Shiozaki et al. 2025). If seawater were the dominant source, sea ice diazotroph assemblages would be expected to show greater similarity to SSW and reflect this variability, rather than the coherent community structure observed in our samples.

In contrast to the diazotrophic assemblages, the overall prokaryotic communities in sea ice and sediments showed pronounced differences. The microbial assemblage in sea ice represents a composite of taxa originating from multiple sources including seawater, sediments, and other input during ice formation (Lund‐Hansen et al. 2024). Unlike diazotrophs, which are strongly biased toward sedimentary habitats, many other microbial groups lack such habitat specificity and are therefore shaped by the full spectrum of microbial environments incorporated into the ice matrix.

We also detected ASVs in SSW belonging to the phylum Thermodesulfobacteriota, the same group found in sediments, with several ASVs occurring in both environments. This finding indicates that a portion of seawater diazotrophs may originate from sedimentary sources. The water column in this region remains well oxygenated and does not develop hypoxic conditions even near the bottom (Nishino et al. 2016), implying that Thermodesulfobacteriota can persist in seawater only within localized anaerobic microhabitats associated with particulate matter. It is therefore highly likely that these taxa are introduced into SSW through sediment resuspension. Given the exceptionally wide and shallow continental shelf of the Arctic Ocean compared to other basins, the contribution of sediment‐derived diazotrophs to seawater communities is likely to be disproportionately large.

## 5 Conclusion

This study demonstrates that diazotrophs detected in sea ice and seawater of the Pacific Arctic likely originate from underlying sediments. It should be noted that the presence of *nifH* does not necessarily indicate active nitrogen fixation. Future work should determine the extent to which sediment‐derived diazotrophs remain metabolically active when transported into environments distinct from their native habitat, and assess their contributions to N cycling across these settings. The Arctic Ocean is undergoing rapid environmental change driven by global warming, and the seafloor is no exception (März et al. 2022; Attard et al. 2024). It is therefore critical to investigate how sediment‐derived diazotrophs respond to these shifts, and how their distribution and activity may be altered under changing Arctic conditions.

## Author Contributions

**Haitian Bo:** investigation, writing – original draft, conceptualization, formal analysis, visualization, writing – review and editing, data curation, methodology, software, validation. **Amane Fujiwara:** investigation, writing – review and editing. **Satoshi Kimura:** investigation, writing – review and editing. **Ziyuan Zhang:** investigation. **Yuri Fukai:** investigation, writing – review and editing. **Masato Ito:** investigation, writing – review and editing. **Koji Hamasaki:** investigation, writing – review and editing, supervision. **Laura Whitmore:** investigation, writing – review and editing. **Marc Oggier:** investigation, writing – review and editing. **Hisatomo Waga:** investigation, writing – review and editing. **Takuhei Shiozaki:** conceptualization, funding acquisition, investigation, writing – review and editing, project administration, supervision, data curation.

## Funding

This work was supported by Japan Society for the Promotion of Science, JP23H05411, JP23K21744, JP24K22347, JP25K03239. Japan Science and Technology Agency, JPMJSP2108. Ministry of Education, Culture, Sports, Science and Technology, ArCSII, ArCSIII.

## Ethics Statement

The authors have nothing to report.

## Conflicts of Interest

The authors declare no conflicts of interest.

## Supporting information


**Table S1:** Nutrient and salinity profile in each sample.
**Table S2:** Depth of seafloor at each station.
**Figure S1:** Vertical variation of nutrient concentrations across sea‐ice layers and underlying seawater.

## Data Availability

The 16S rRNA and nifH gene sequence data generated in this study are available from the National Center for Biotechnology Information (NCBI) under BioProject accession number PRJNA1371680.
