# Supplementary material for: Sediments as Potential Sources of Non‐Cyanobacterial Diazotrophs in Arctic Sea Ice and Seawater
Source: Environ Microbiol Rep. 2026 Jun 4;18(3):e70372. doi: 10.1111/1758-2229.70372 (PMC13238701; doi:10.1111/1758-2229.70372)
Supplement: Supplementary file 1 — Table S1: Nutrient and salinity profile in each sample. Table S2: Depth of seafloor at each station. Figure S1: Vertical variation of nutrient concentrations across sea‐ice layers and underlying seawater. [file EMI4-18-e70372-s001.docx]

Supplementary Information for

Sediments as potential sources of non-cyanobacterial diazotrophs

in Arctic sea ice and seawater

H. Bo, Z. Zhang, A. Fujiwara, Y. Fukai, M. Ito, S. Kimura, M. Oggier, H. Waga,

L. Whitmore, K. Hamasaki, T. Shiozaki

Correspondence to: shiozaki@g.ecc.u-tokyo.ac.jp

**This PDF file includes:**

Table S1 and S2

Fig. S1

Table S1 Nutrient and salinity profile in each sample

| Station | Sample Source | NO_3_^-^ [µΜ] | NO_2_^-^ [µΜ] | NH_4_^+^ [µΜ] | PO_4_^3-^ [µΜ] | N/P | Salinity |
| --- | --- | --- | --- | --- | --- | --- | --- |
| MR21-3 | SSW | 0.19 | 0.03 | 0.63 | 0.54 | 1.57 | 30.19 |
| MR21-9 | SSW | UDL | UDL | 0.04 | 0.48 | 0.17 | 25.89 |
| MR21-14 | SSW | 0.05 | UDL | UDL | 0.44 | 0.16 | 28.46 |
| MR21-17 | SSW | UDL | UDL | 0.09 | 0.51 | 0.22 | 26.32 |
| MR21-22 | SSW | UDL | 0.01 | 0.08 | 0.52 | 0.21 | 29.78 |
| MR21-24 | SSW | 0.03 | UDL | 0.05 | 0.59 | 0.14 | 28.35 |
| MR21-28 | SSW | UDL | 0.01 | UDL | 0.50 | 0.10 | 28.82 |
| MR21-31 | SSW | 0.39 | 0.02 | 0.27 | 0.53 | 1.29 | 30.65 |
| MR21-33 | SSW | 0.45 | 0.02 | 0.69 | 0.58 | 1.99 | 30.81 |
| MR21-35 | SSW | 0.10 | 0.01 | 0.08 | 0.53 | 0.36 | 30.10 |
| MR21-38 | SSW | 0.06 | 0.02 | 0.36 | 0.60 | 0.74 | 30.03 |
| MR21-42 | SSW | 0.32 | 0.07 | 0.49 | 0.65 | 1.36 | 29.06 |
| MR21-45 | SSW | 0.04 | 0.01 | 0.12 | 0.36 | 0.47 | 28.85 |
| MR21-54 | SSW | 1.62 | 0.06 | 2.09 | 0.84 | 4.49 | 30.47 |
| MR21-58 | SSW | 1.75 | 0.05 | 0.68 | 0.71 | 3.48 | 30.97 |
| MR21-62 | SSW | 7.76 | 0.06 | 0.84 | 1.11 | 7.80 | 32.10 |
| MR21-9 | Bottom-5m | 12.59 | 0.14 | 1.06 | 1.72 | 8.04 | 32.75 |
| MR21-14 | Bottom-5m | 12.42 | 0.34 | 4.12 | 2.07 | 8.17 | 32.74 |
| MR21-24 | Bottom-5m | 11.44 | 0.21 | 2.82 | 1.84 | 7.85 | 32.80 |
| MR21-28 | Bottom-5m | 11.95 | 0.55 | 4.80 | 2.08 | 8.33 | 32.66 |
| MR21-31 | Bottom-5m | 10.99 | 0.17 | 6.20 | 1.97 | 8.83 | 32.63 |
| MR21-33 | Bottom-5m | 7.75 | 0.15 | 6.12 | 1.70 | 8.24 | 32.33 |
| MR21-35 | Bottom-5m | 6.56 | 0.41 | 2.91 | 1.46 | 6.75 | 32.32 |
| MR21-38 | Bottom-5m | 1.42 | 0.22 | 3.93 | 1.14 | 4.90 | 31.32 |
| MR21-42 | Bottom-5m | 11.73 | 0.18 | 4.83 | 1.72 | 9.71 | 32.35 |
| MR21-45 | Bottom-5m | 19.94 | 0.23 | 9.41 | 2.71 | 10.91 | 32.86 |
| MR21-54 | Bottom-5m | 13.51 | 0.12 | 6.97 | 2.06 | 9.99 | 32.53 |
| MR21-58 | Bottom-5m | 18.96 | 0.16 | 4.50 | 2.05 | 11.50 | 32.74 |
| MR21-62 | Bottom-5m | 10.70 | 0.08 | 1.80 | 1.44 | 8.72 | 32.25 |
| BRW01 Top | Ice Top | 0.82 | 0.04 | 0.52 | 0.26 | 5.32 | 4.90 |
| BRW01 Mid | Ice Mid | 0.47 | UDL | 0.39 | 0.09 | 9.06 | 3.10 |
| BRW01 Bot | Ice Bot | 2.71 | 0.16 | 0.92 | 1.26 | 3.00 | 5.80 |
| BRW01 SWBI | SWBI | 9.86 | 0.07 | 1.94 | 1.46 | 8.14 | 32.80 |
| BRW02 Top | Ice Top | 0.91 | 0.14 | 1.34 | 0.55 | 4.35 | 4.30 |
| BRW02 Mid | Ice Mid | 0.60 | UDL | 0.61 | 0.16 | 7.61 | 4.00 |
| BRW02 Bot | Ice Bot | 3.16 | 0.11 | 0.75 | 1.02 | 3.92 | 5.30 |
| BRW02 SWBI | SWBI | 12.04 | 0.09 | 2.25 | 1.69 | 8.49 | 32.90 |
| BRW03 Top | Ice Top | 0.40 | UDL | 0.56 | 0.17 | 5.70 | 6.80 |
| BRW03 Mid | Ice Mid | 0.55 | UDL | 0.61 | 0.08 | 15.21 | 4.10 |
| BRW03 Bot | Ice Bot | 13.76 | 0.21 | 4.45 | 4.63 | 3.98 | 8.00 |
| BRW03 SWBI | SWBI | 12.04 | 0.10 | 2.57 | 1.68 | 8.74 | 32.80 |
| BRW04 Top | Ice Top | 1.37 | 0.32 | 0.96 | 1.17 | 2.26 | 4.90 |
| BRW04 Mid | Ice Mid | 0.55 | UDL | 0.62 | 0.13 | 8.89 | 4.30 |
| BRW04 Bot | Ice Bot | 4.50 | 0.13 | 1.50 | 1.52 | 4.03 | 6.40 |
| BRW04 SWBI | SWBI | 8.07 | 0.06 | 1.84 | 1.31 | 7.63 | 32.80 |
| BRW05 Top | Ice Top | 1.63 | 0.45 | 1.68 | 1.63 | 2.31 | 4.70 |
| BRW05 mid | Ice Mid | N/A | N/A | N/A | N/A | N/A | N/A |
| BRW05 bot | Ice Bot | N/A | N/A | N/A | N/A | N/A | N/A |
| BRW05 SWBI | SWBI | 5.18 | 0.04 | 2.34 | 0.99 | 7.62 | 33.20 |
| BRW07 top | Ice Top | N/A | N/A | N/A | N/A | N/A | N/A |
| BRW07 Mid | Ice Mid | 1.66 | 0.04 | 1.19 | 0.25 | 11.68 | 5.20 |
| BRW07 Bot | Ice Bot | 2.37 | 0.03 | 0.64 | 0.45 | 6.68 | 5.50 |
| BRW07 SWBI | SWBI | N/A | N/A | N/A | N/A | N/A | N/A |

UDL: under detection limit, N/A: not available

Table S2 Depth of seafloor at each station

| Station | Depth [m] |
| --- | --- |
| MR21-3 | 101 |
| MR21-9 | 67 |
| MR21-11 | 1022 |
| MR21-14 | 11 |
| MR21-17 | 1003 |
| MR21-19 | 642 |
| MR21-22 | 399 |
| MR21-24 | 20 |
| MR21-28 | 14 |
| MR21-31 | 22 |
| MR21-33 | 22 |
| MR21-35 | 18 |
| MR21-39 | 8 |
| MR21-42 | 11 |
| MR21-45 | 9 |
| MR21-54 | 15 |
| MR21-58 | 21 |
| MR21-62 | 33 |


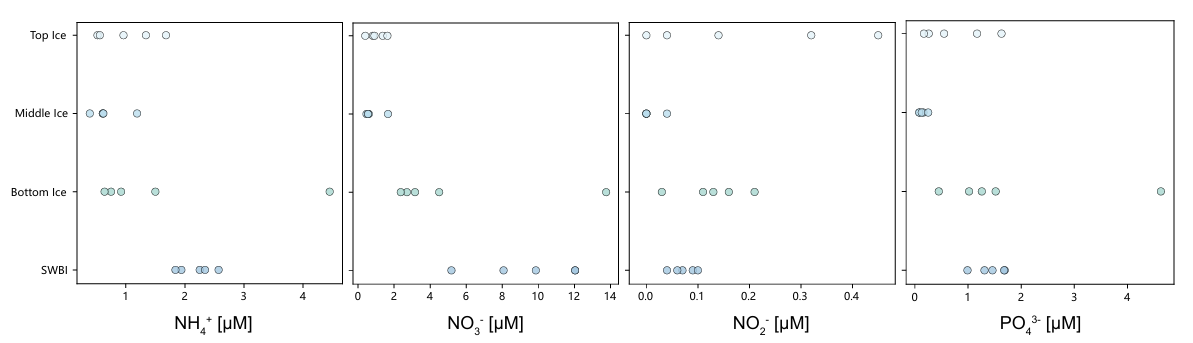


Fig. S1 Vertical variation of nutrient concentrations across sea-ice layers and underlying seawater.
